# Climate Impacts on Sea Turtle Breeding Phenology in Greece and Associated Foraging Habitats in the Wider Mediterranean Region

**DOI:** 10.1371/journal.pone.0157170

**Published:** 2016-06-22

**Authors:** Samir H. Patel, Stephen J. Morreale, Vincent S. Saba, Aliki Panagopoulou, Dimitris Margaritoulis, James R. Spotila

**Affiliations:** 1 Department of Biodiversity, Earth and Environmental Science, Drexel University, Philadelphia, PA, 19104, United States of America; 2 Cornell University, Department of Natural Resources, Ithaca, NY, 14850, United States of America; 3 NOAA NMFS NEFSC, c/o Geophysical Fluid Dynamics Laboratory, Princeton University Forrestal Campus, Princeton, NJ, 08544, United States of America; 4 ARCHELON, the Sea Turtle Protection Society of Greece, 104 32 Athens, Greece; Deakin University, AUSTRALIA

## Abstract

Sea turtles are vulnerable to climate change impacts in both their terrestrial (nesting beach) and oceanic habitats. From 1982 to 2012, air and sea surface temperatures at major high use foraging and nesting regions (n = 5) of loggerhead turtles (*Caretta caretta*) nesting in Greece have steadily increased. Here, we update the established relationships between sea surface temperature and nesting data from Zakynthos (latitude: 37.7°N), a major nesting beach, while also expanding these analyses to include precipitation and air temperature and additional nesting data from two other key beaches in Greece: Kyparissia Bay (latitude: 37.3°N) and Rethymno, Crete (latitude: 35.4°N). We confirmed that nesting phenology at Zakynthos has continued to be impacted by breeding season temperature; however, temperature has no consistent relationship with nest numbers, which are declining on Zakynthos and Crete but increasing at Kyparissia. Then using statistically downscaled outputs of 14 climate models assessed by the Intergovernmental Panel on Climate Change (IPCC), we projected future shifts in nesting for these populations. Based on the climate models, we projected that temperature at the key foraging and breeding sites (Adriatic Sea, Aegean Sea, Crete, Gulf of Gabès and Zakynthos/Kyparissia Bay; overall latitudinal range: 33.0°—45.8°N) for loggerhead turtles nesting in Greece will rise by 3–5°C by 2100. Our calculations indicate that the projected rise in air and ocean temperature at Zakynthos could cause the nesting season in this major rookery to shift to an earlier date by as much as 50–74 days by 2100. Although an earlier onset of the nesting season may provide minor relief for nest success as temperatures rise, the overall climatic changes to the various important habitats will most likely have an overall negative impact on this population.

## Introduction

Although warming of the oceans is three times slower than warming of air temperature over land, marine species are shifting distributions and phenology at a greater rate than species in terrestrial systems [1]. The Mediterranean Sea is a somewhat uniquely diverse system, with subtropical species residing in southern waters, and temperate species thriving in the north [2]. Although a relatively small basin, comprising 0.82% surface area and 0.32% volume of the world’s total ocean, an estimated 4–18% of the earth’s marine species permanently reside in the Mediterranean Sea [2]. In addition, over a quarter of the extant species are endemic to the Mediterranean, including a sea grass species, *Posidonia oceanica*, which is critical in maintaining such high levels of biodiversity [2]. High biodiversity notwithstanding, anthropogenic impacts (pollution, overfishing, habitat destruction, and species introductions) are reaching a climax in the Mediterranean region, causing extensive environmental damage [3]. Coupled with these direct impacts are projections of a continuously changing climate, which means more biodiversity loss may be expected in the future.

Recently the loggerhead turtle (*Caretta caretta*) subpopulation in the Mediterranean Sea was downgraded to Least Concern by the IUCN [4]; however, substantial declines in nest numbers have been reported at major nesting sites in the region [5,6]. In the Eastern Mediterranean, loggerhead turtles are found across almost the entire basin’s latitudinal range, ~31.0°- 45.6° N; but close to 25% of all recorded annual nesting activity occurs on three Greek beaches, and foraging is localized to benthic environments in five sites [7–12]. Consequently, shifts in environmental conditions at a regional scale, or even within a single nesting area, may have a severe impact on the overall survival of this species [13], but at least likely will greatly impact the Mediterranean subpopulation.

There are many potential impacts of climate change on sea turtles, including loss of nesting habitat due to sea level rise and decline in hatching success with incubation temperatures reaching lethal limits [14–16]. Also, effects of sea surface temperature (SST) causing shifts in nesting patterns of sea turtles have already been demonstrated [17–22]. In addition, water temperature affects the internesting intervals of sea turtles [23], and air temperature (T_a_) and precipitation in nesting beach habitats affect sex ratios of hatchlings and survival of eggs [24–26].

In the Mediterranean Sea, Greece is home to the largest population of nesting loggerheads, with Zakynthos Island, Kyparissia Bay and Rethymno, Crete ranked 1, 2 and 3 in order of nest numbers per season. Recent averages (2008–2012) have been reported to be 902, 600 and 184 nests year^-1^ at the three sites, respectively [4]. At Zakynthos Island, warming of sea surface temperature near the breeding sites is associated with an earlier date of first adult female emergence, reduced clutch sizes, and increased hatching success [27]. In addition, because gender of marine turtles is determined by the temperature of incubation [28–30], increasing air and sand temperatures may affect sex ratios of populations [24,26,31–35].

In general, availability of prey resources in sea turtle foraging habitats also is critical to reproductive success [19,36–38]. Females depend on specific amounts of food resources for vitellogenesis (formation of yolk in eggs) prior to migrating to the nesting beach. Less food could result in delayed returns; thus, sea turtles will nest less frequently if food availability continues to be limited [19,36,37,39]. Indeed, variability in primary and secondary production in sea turtle foraging areas also contributes substantially to nesting abundance [12,19,21,40].

It is likely that variability in the distribution of prey resources in the Mediterranean results in a fitness dichotomy in which loggerhead turtles foraging in northern habitats are larger and produce larger clutch sizes than loggerheads foraging in southern habitats [12,41,42]. Loggerheads in the Mediterranean feed on slow-moving benthic organisms associated with (or typically found in) sea grass beds [43,44]. These sea grass beds, specifically composed of *Posidonia oceanica*, an endemic species of the Mediterranean, are critical habitats contributing to the overall biodiversity of the region [45]. Importantly, water temperature plays a role in the physiology and overall survival of sea grasses [46,47], and rising temperatures associated with climate change likely will have a profound effect on the survival of sea grass meadows [48] and possibly the future of Mediterranean loggerhead prey availability.

Here we examined the effects of climate change on loggerhead turtles in the Eastern Mediterranean Sea. The objective of this study was to identify how future changes to key climatic variables would influence nesting and foraging of loggerheads in this region. To that end, we first investigated correlations between SST, T_a_ and precipitation and existing nesting data for loggerhead turtles from Greece. Then, we used global climate models assessed by the IPCC to project the environmental conditions of the high-use areas for Greek loggerheads within the Eastern Mediterranean Sea through the 21^st^ century. Finally, based on historical trends, we projected the impacts of future climatic changes on this subpopulation of loggerheads.

## Methods

### Nesting and foraging sites

Nesting and mating information for Greece were derived from data reported by Margaritoulis et al. [5–7], Margaritoulis [8], Margaritoulis and Rees [49,50], Mazaris et al. [22,27] and Schofield et al. [51,52]. Because access to ARCHELON monitoring data was limited to those data in the previously listed publications, for Crete (10.8 km of nesting beach), nesting data spanned from 1990–2004; for Kyparissia (7.3 km of nesting beach), nesting data spanned from 1984–2000; and for Zakynthos (5.5 km of nesting beach), nesting data spanned from 1984–2009. From these publications, we designated the mating season to be April—June, and focused our analyses on Zakynthos when data were not available from Kyparissia and Rethymno, as Zakynthos is home to the highest nesting aggregation and the most complete dataset. In terms of Zakynthos nesting, timely and regular surveys permitted the precise recording of the first emergence (and nest) date at Sekania (the most turtle-frequented beach sector, concentrating more than 50% of all nesting in Zakynthos) during most seasons [8]. In five seasons (1986, 1998–2001) the start of the nesting activity was missed because female tracks were already present on the beach at first survey [8]. During these five seasons the dates of the first emergence, and nest, were estimated either by the external appearance of the tracks or by the date of the first hatch of the season, from which the mean incubation duration of the season was subtracted [8]. Monitoring protocols in acquiring the nesting data used in this study were consistent between all three sites.

**Fig 1:**
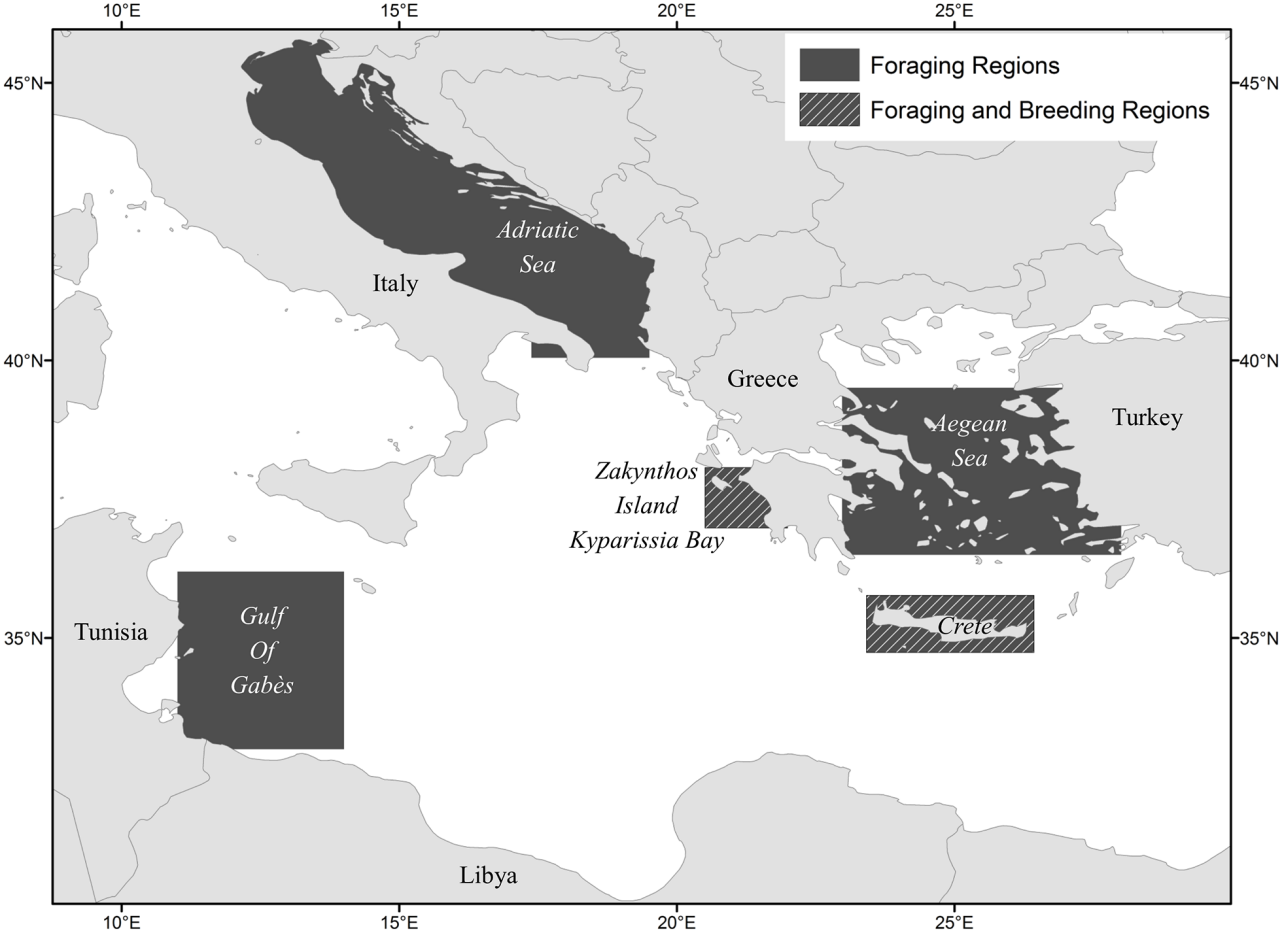
Eastern Mediterranean Sea with indication of the 5 high use sites for loggerheads.

We selected five high-use regions for loggerheads in the Mediterranean Sea (Adriatic, Aegean, Crete, Gulf of Gabès, and Zakynthos/Kyparissia Bay), based on the combined results of telemetry studies independently conducted at the three focal nesting beaches by Schofield et al. [10,51], Zbinden at el. [41], Panagopoulou [53], Backof [54] and Patel et al. [11,12] (Fig 1). All five regions are home to foraging aggregations, and Zakynthos/Kyparissia and Crete have seasonal internesting cohorts. From Zakynthos, Schofield et al. [10,51] and Zbinden et al. [41] reported that the majority of postnesting loggerheads migrated into the Adriatic Sea, the Gulf of Gabès, the Aegean Sea, and stayed resident adjacent to Zakynthos, while the majority of internesting turtles remained within and adjacent to Zakynthos and western Peloponnese waters. From Kyparissia, Backof [54] tracked two postnesting loggerheads, with one migrating into the Adriatic Sea, along with internesting loggerheads that remained within Kyparissia Bay. From Rethymno, Patel et al. [11,12] tracked postnesting loggerheads that migrated into the Aegean Sea, remained resident in Cretan waters, and travelled to the Gulf of Gabès; while Panagopoulou [53] tracked internesting turtles that remained along the north coast of Crete.

### Current and historical environmental data

During the 2012 nesting season in Rethymno, we measured beach temperatures using six iButtons (Maxim Integrated, San Jose, CA). Data collection was approved and assisted by ARCHELON, the Sea Turtle Protection Society of Greece, which has a permit for research work in this area. The iButtons have a temperature resolution of 0.5°C. These data loggers were calibrated and remained accurate throughout the deployment, and the variability between iButtons was less than 0.5°C. We confirmed this by calculating the average temperature for each data logger across 5 days before and after the sampling period with each logger collecting data within the same environment. To measure sand temperatures along this predominantly north-facing beach, we placed one iButton at the surface of the sand and another at a depth of 50 cm, equivalent to the depth of loggerhead nests [9]. Each iButton measured the temperature every 60 minutes from May 21—Aug 1, encompassing the time period during which ~99% of monitored nests were laid. The iButton was encased in a film canister punctured with several holes to protect the device from outside elements. Based on historical nesting records identifying the beach sectors with the most nests/km/season [5,50], we placed iButtons at three sites of high nesting activity halfway between the high tide line and the back of the beach (Location 1: 35.3697° N, 24.51518° E, Location 2:35.3821° N, 24.5818° E, Location 3: 35.3911° N, 24.6085° E). Location 1, the farthest west, was 6.2 km from location 2 and 8.8 km from location 3, the farthest east. Locations 2 and 3 were on beach sectors patrolled at night; all three locations were on sectors that were patrolled during the mornings in order to mark and count the previous night’s nesting activity.

For the focal nesting and foraging regions, we extracted monthly SST values with 1° resolution from 1982 to 2012 from the NOAA NCEP EMC CMB Global Reynolds and Smith OI version 1 dataset [55]. We also obtained historical precipitation data (1982–2000) from the Global Precipitation Climatology Centre (GPCC) and historical air temperature data (1982–2012) from weather stations at the international airports of Laganas on Zakynthos, Heraklion on Crete, and Kalamata in southern Peloponnesus. Climatological data from nearby airports were later used to downscale from global to regional-scale models, as has been done successfully in other studies [20,24,34]. The Laganas airport (37.75° N, 20.88° E; elevation: 3 m) is ~3 km from the nesting beaches of Zakynthos; Heraklion airport (35.34° N, 25.17° E; elevation: 39 m) is ~60 km from the nesting beaches of Rethymno; and the Kalamata airport (37.07° N, 22.03° E; elevation: 8 m) is ~40 km from the nesting beaches of Kyparissia Bay.

### Future climate model data

We obtained historical (1984–2005) and future (2006–2100) climate change projections for the 5 high-use regions from global climate models developed for the Intergovernmental Panel on Climate Change (IPCC) fifth assessment report (AR5) and for the World Climate Research Programme’s Coupled Model Intercomparison Project phase 5 (CMIP5) under the representative concentration pathway (RCP) 8.5 long-lived greenhouse gas emissions scenario. RCP 8.5 is the highest expected level of greenhouse gas emissions. For SST, we used 13 global climate models, and for T_a_ and precipitation, we used 14 atmospheric models. All models used in this study are listed in the supplementary information (S1 Appendix).

To statistically downscale the models from a global to a regional scale, we bias-corrected all climate model outputs (both historical and RCP 8.5 projections) using the mean and standard deviation of observed data from 1984–2005 [20]. As a result of this process, we determined the projected change in temperature/precipitation for each model in each region. With this method, first we calculated the mean and standard deviations of monthly observed data and the historical runs from the climate models for the years 1984–2005. Then, we calculated a mean bias correction factor by dividing the average monthly mean of the observed data by the average monthly mean of the same month from the historical climate model data. We calculated SD bias correction factors by dividing the corresponding SD values. Next, we multiplied the mean bias correction factor for each month by the corresponding monthly mean from the historical and future climate model projections on an annual basis. Then, we subtracted this mean bias-corrected value from the corresponding average monthly mean from the observed data. We multiplied this new value by the SD bias correction factor of the corresponding month, and finally, we added this value to the average monthly mean from the observed data. This resulted in the bias-corrected historical climate model output having the same mean and SD as the observed data for the same time period (1984–2005). These same bias-correction methods were applied to the RCP 8.5 future projections.

### Data analyses

To test for significance of change in SST, T_a_ and precipitation through time, we performed a simple linear regression (statistical significance set at a level of 0.05). We used linear regression models to test the statistical significance of the relationship between each environmental variable (precipitation, T_a_ and SST) and patterns in nesting data [22;24]. Linear regression models in this study were identified as valid based on analysis of the residuals. Statistical significance was tested at the 95% confidence interval. To make projections on how the start of the nesting season would shift as SST and T_a_ changed, we used models calculated from the linear trend lines of relationships between SST and T_a_ and day of first female emergence at Zakynthos.

## Results

Beach temperatures at Rethymno varied among the three locations monitored by iButtons during the 2012 nesting season. Sand surface temperatures reached a high of 61.5°C and a low of 13.0°C, while nest depth temperatures were much less variable (range = 23.5–31.0°C) (S2 Appendix). Beginning on July 7, temperatures at the 50 cm depth at location 1 remained consistently above 29.5°C, which is above the calculated female-producing pivotal temperature for loggerheads at Kyparissia Bay [30]. At locations 2 and 3, female-producing temperatures were not observed consistently until July 27.

From the historical data over three decades, there was a steady and statistically significant upward trend in SST at Crete during consecutive mating seasons (April—June), increasing from $\bar{\text{x}}$ = 19.0°C in 1982, to $\bar{\text{x}}$ = 20.2°C in 2012 (R^2^ = 0.477, p < 0.01, m = 0.04) (S3 Appendix). The lowest SST during those months occurred in 1987 (18.2°C), and the highest occurred in 2012. Mean SST over the 30 year period at Crete, the more southern site by ~2°, was slightly warmer (0.4°C) than at Zakynthos and Kyparissia. Nevertheless, there was a similar rise in SST over the course of the same time period at Zakynthos/Kyparissia Bay (R^2^ = 0.370, p < 0.01, m = 0.04). The coldest mating season at Zakynthos/Kyparissia occurred in 1991 (17.9°C), and the warmest occurred in 2003 (20.0°C). Using Zakynthos nesting data from 1984–2002, Mazaris et al. [27] found a significant relationship between the rise in SST during the mating season and an earlier date of first female emergence during the season. Using updated nesting data from Zakynthos (1984–2009), we calculated a similar significant correlation (p < 0.001, R^2^ = 0.830, y = -12.157(x) + 378.97), with females continuing to emerge onto the nesting beach earlier in the season (S4 Appendix).

**Fig 2:**
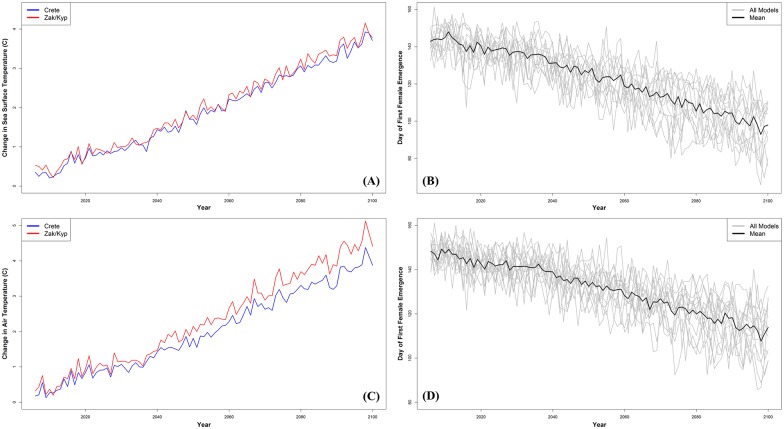
Climate model projections of mating period SST and T_a_ and corresponding projections of the day of first female emergence through the 21^st^ century. (A) Means of the projected changes in SST from 13 climate models (RCP 8.5) during the mating season for Crete and Zakynthos/Kyparissia. (B) Projections of the day of first female emergence through 2100 based on climate model estimations of the increase in SST during the mating season at Zakynthos Island. (C) Means of the projected changes in T_a_ from 14 atmospheric models (RCP 8.5) during the mating season for Crete and Zakynthos/Kyparissia. (D) Projections of the day of first female emergence through 2100 based on atmospheric model estimations on the increase in T_a_ during the mating season at Zakynthos Island.

Based on this same relationship, future nesting season patterns were projected using statistically downscaled climate change models. From the means of these climate model projections for the three sites, we calculated that mean SST during the mating season will rise by between 2.4–6.0°C in all three nesting areas by 2100 (Fig 2A). Using the bias-corrected data of climate projections based on the mean and SD of the observed data, we also calculated that the start of the nesting season in Zakynthos will shift to an earlier date by 52.5 ± 12.0 days (mean ± SD), with a range of 39.6–74.8 days by 2100 (Fig 2B). This shift would advance the day of first female emergence from late May to between mid-March and Mid-April.

**Fig 3:**
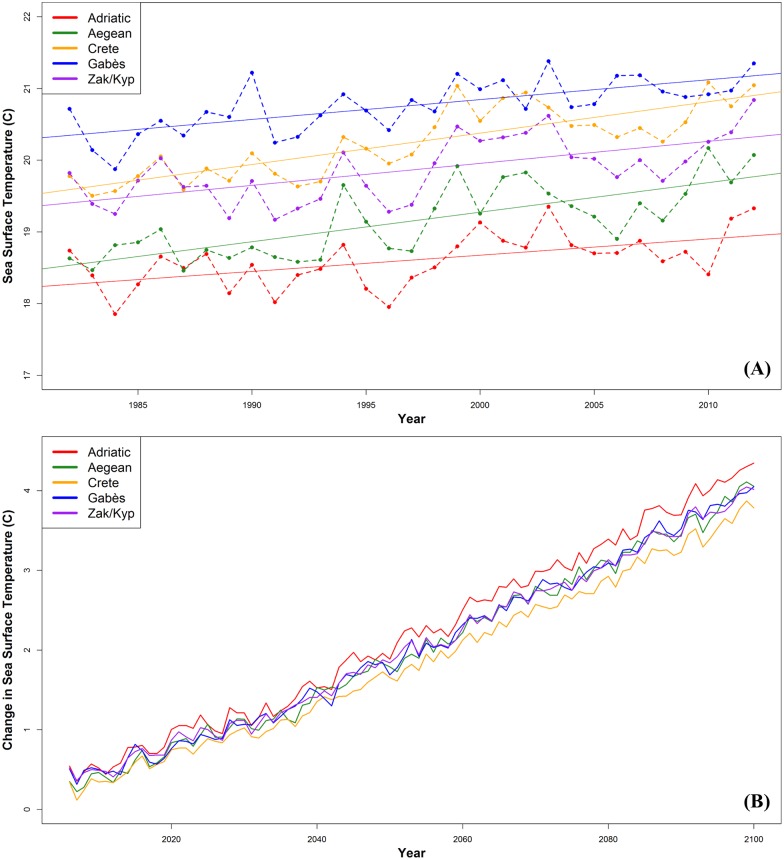
Observed and future projections of mean annual SST for the five high use regions. (A) Mean annual SST at the 5 high use areas for loggerheads in the Mediterranean. Solid lines are the linear trend lines (Adriatic R^2^ = 0.311; Aegean R^2^ = 0.560; Crete R^2^ = 0.674; Gabès R^2^ = 0.474; Zak/Kyp R^2^ = 0.393). (B) Means of the projected changes in annual SST from 13 climate models (RCP 8.5) for the 5 high use sites.

**Fig 4:**
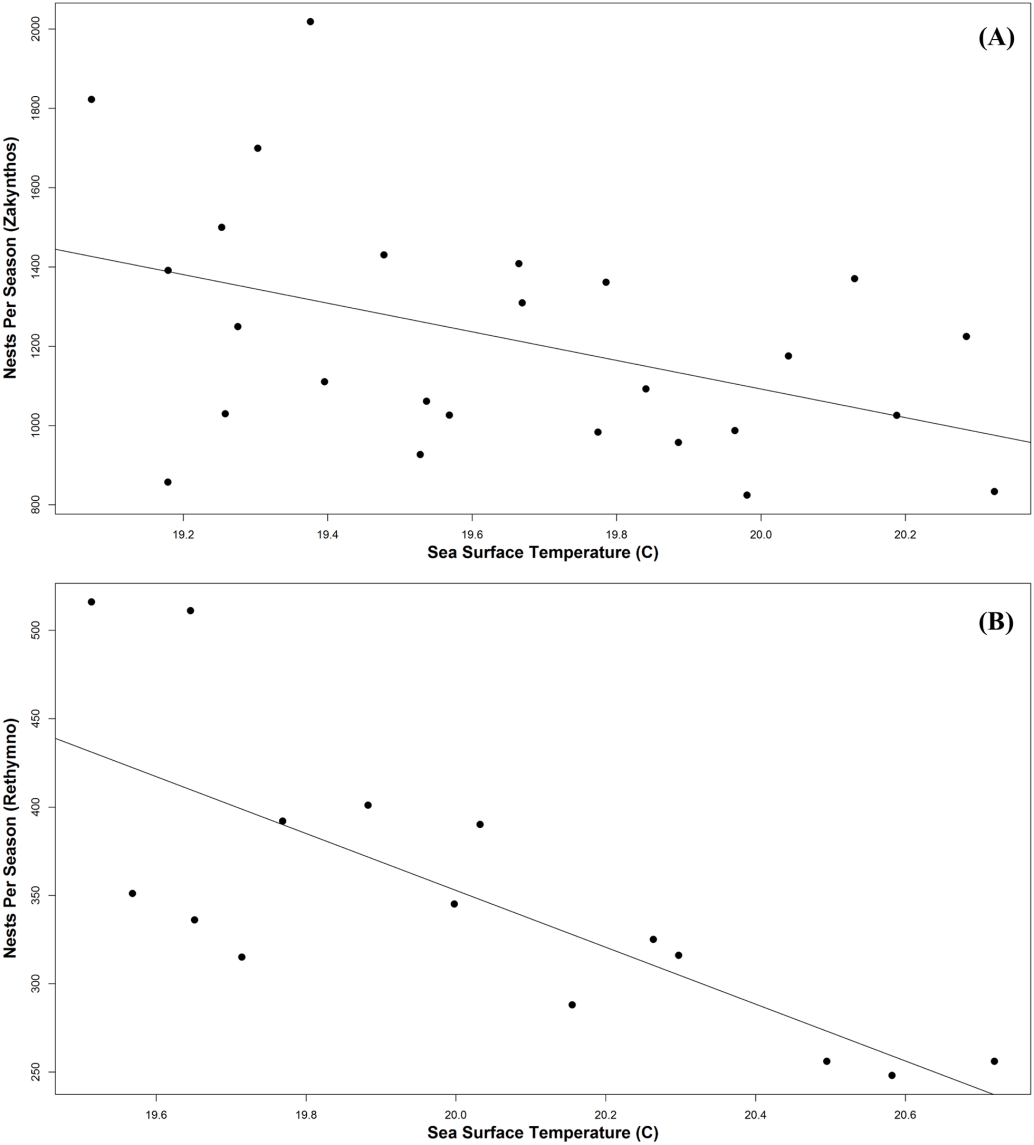
Relationship between number of nests per season and foraging site SST two years prior. (A) Relationship between number of nests per season at Zakynthos (1984–2009) and the mean annual SST at the 5 foraging sites 2 years prior. Solid line is the linear trend line (R^2^ = 0.190). (B) Relationship between number of nests per season at Rethymno (1990–2004) and the mean annual SST at the foraging sites (Gulf of Gabès, Aegean Sea and Crete) 2 years prior. Solid line is the linear trend line (R^2^ = 0.572).

Within the foraging areas, the mean annual SST from 1982–2012 varied by region, with the Adriatic Sea being the coldest ($\bar{\text{x}}$ = 18.6°C, range = 17.9–19.4°C), and the Gulf of Gabès the warmest ($\bar{\text{x}}$ = 20.8°C, range = 19.9–21.4°C) (Fig 3A). As observed at the nesting beaches, there was a significant and steadily increasing trend in mean annual SST from 1982 through 2012 at the five selected foraging sites around the Mediterranean. SST increased 0.6°C (R^2^ = 0.311, p = 0.04, m = 0.02) in the Adriatic, 1.4°C (R^2^ = 0.560, p < 0.01, m = 0.04) in the Aegean, 1.3°C (R^2^ = 0.674, p < 0.01, m = 0.04) around Crete, 0.6°C (R^2^ = 0.474, p < 0.01, m = 0.03) in the Gulf of Gabès, and 1.0°C (R^2^ = 0.393, p < 0.01, m = 0.03) around Zakynthos/Kyparissia. Extending the trend, the bias-corrected climate models projected a steady increase in the mean annual SST of 2.1–6.5°C (m = 0.04) for all regions for the years 2013–2100 (Fig 3B). For Zakynthos, using nesting data from 1984–2007, Mazaris et al. [22] reported a significant relationship between the temperature at the foraging site two years prior to the nesting season and an observed decline in nests per season. We calculated that this correlation continued (p = 0.03, df = 25), using updated nesting data from 1984–2009 (Fig 4A). We also calculated a similar trend in Rethymno, from 1990–2004, with a 1.2°C increase in temperature at the foraging sites (Aegean Sea, Crete, and Gulf of Gabès) corresponding to a decrease of 260 nests per season (p < 0.01, df = 14) (Fig 4B). In Kyparissia, we did not detect a significant correlation between foraging site SST and total nests per season (p = 0.7; df = 16).

Over the course of the past 30 years in all regions, there were increases in mean SST in the month of August, which is on average the hottest month of the year in all five regions (Adriatic: 1.6°C, R^2^ = 0.046, m = 0.02, p = 0.25; Aegean: 1.8°C, R^2^ = 0.555, p < 0.01, m = 0.07; Crete: 1.5°C, R^2^ = 0.499, p < 0.01, m = 0.05; Gabès: 1.4°C, R^2^ = 0.190, m = 0.03, p = 0.01; Zak/Kyp: 1.3°C, R^2^ = 0.251, p < 0.01, m = 0.04) (S5A Appendix). The overall mean SST in August varied by region, with the Gulf of Gabès, the southernmost area, being the warmest ($\bar{\text{x}}$ = 27.1°C, range = 25.9–28.6°C), and the Aegean Sea the coldest ($\bar{\text{x}}$ = 24.7°C, range = 23.2–26.3°C). By 2100, August SSTs in each region are expected to increase by (mean ± SD) 4.4 ± 1.3°C (m = 0.05) (S5B Appendix). This projected increase in temperature has potentially severe implications for the survival of sea grass in the key foraging sites for loggerheads in the Mediterranean.

At the nesting sites during the mating season, T_a_ rose significantly in Zakynthos (R^2^ = 0.467; p < 0.01, m = 0.08) and Kyparissia Bay (R^2^ = 0.252; p < 0.01, m = 0.05) between 1982 and 2009, but not in Rethymno (R^2^ = 0.133; p = 0.06, m = 0.03) (S6 Appendix). We calculated that there was no significant relationship between T_a_ and total nests within the same season (Zakynthos: p = 0.3, df = 25; Kyparissia: p = 0.5, df = 16; Rethymno: p = 1.0, df = 14). There was a significant relationship between T_a_ during the mating season and the date of first female emergence in Zakynthos (p < 0.01, df = 25, R^2^ = 0.705; y = -6.8327(x) + 286.35) (S7 Appendix). The bias corrected climate models project that T_a_ at the nesting sites during the mating season will increase by (mean ± SD) 4.1 ± 1.3°C (m = 0.04) by 2100 (Fig 2C). Based on the previous equation and the projected increase in T_a_, the date of first female emergence will shift earlier by (mean ± SD) 35.5 ± 11.7 days (range = 16.8–50.6 days) by 2100 (Fig 2D).

According to the GPCC, monthly average precipitation from 1982–2000 for all 5 regions was 64.6 mm month^-1^ (± 56.6). November was the wettest month and June was the driest. In all regions, there was no significant change in mean annual precipitation from 1982–2000 (p = 0.5, df = 18). At Zakynthos, Kyparissia, and Crete during the nesting season, June—August, rainfall averaged from 0.13–10.4 mm month^-1^. During the mating season (April—June), rainfall averaged from 2.0–51.9 mm month^-1^. We calculated that annual rainfall at the foraging sites two years prior to the nesting season did not have a significant impact on the number of nests at each site (Zakynthos: p = 0.5, df = 18; Kyparissia: p = 0.6, df = 16; Rethymno: p = 0.1, df = 12). In Zakynthos specifically, we calculated that annual rainfall at the foraging sites two years prior to the nesting season did not have a significant impact on clutch size or the start of the nesting season (clutch size: p = 0.5, df = 18; phenology: p = 0.06; df = 18); and rainfall during the nesting season did not have a significant impact on hatchling success or hatchling emergence success (hatchling success: p = 0.2, df = 16; hatchling emergence success: p = 0.2; df = 16). The bias corrected climate models project that by 2100, rainfall will decline at Crete by as much a 20.3 mm month^-1^ (m = -0.03) and at Zakynthos and Kyparissia by 28.4 mm month^-1^ (m = -0.09).

## Discussion

Sea surface temperatures and air temperatures over land in the Mediterranean region are steadily rising. These and other important shifts in environmental features at the breeding and foraging sites have a significant effect on the timing, quantity, and quality of loggerhead nesting in the Mediterranean Sea [22,27,56]. We found that both a rise in SST and T_a_ at the breeding sites during the mating season corresponded to an earlier start to the nesting season in Zakynthos. It is unclear whether SST or T_a_ plays the stronger role in prompting nesting, as sea turtles demonstrate many behavioral strategies in order to find more suitable temperature, for example residing in warmer spots within the water [57,58], or breaching the surface to bask [59]. Regardless, it is apparent that warmer temperatures, both air and sea, result in earlier nesting seasons and our projections suggest that the nesting season could shift earlier by as much as 74 days by end of this century.

**Fig 5:**
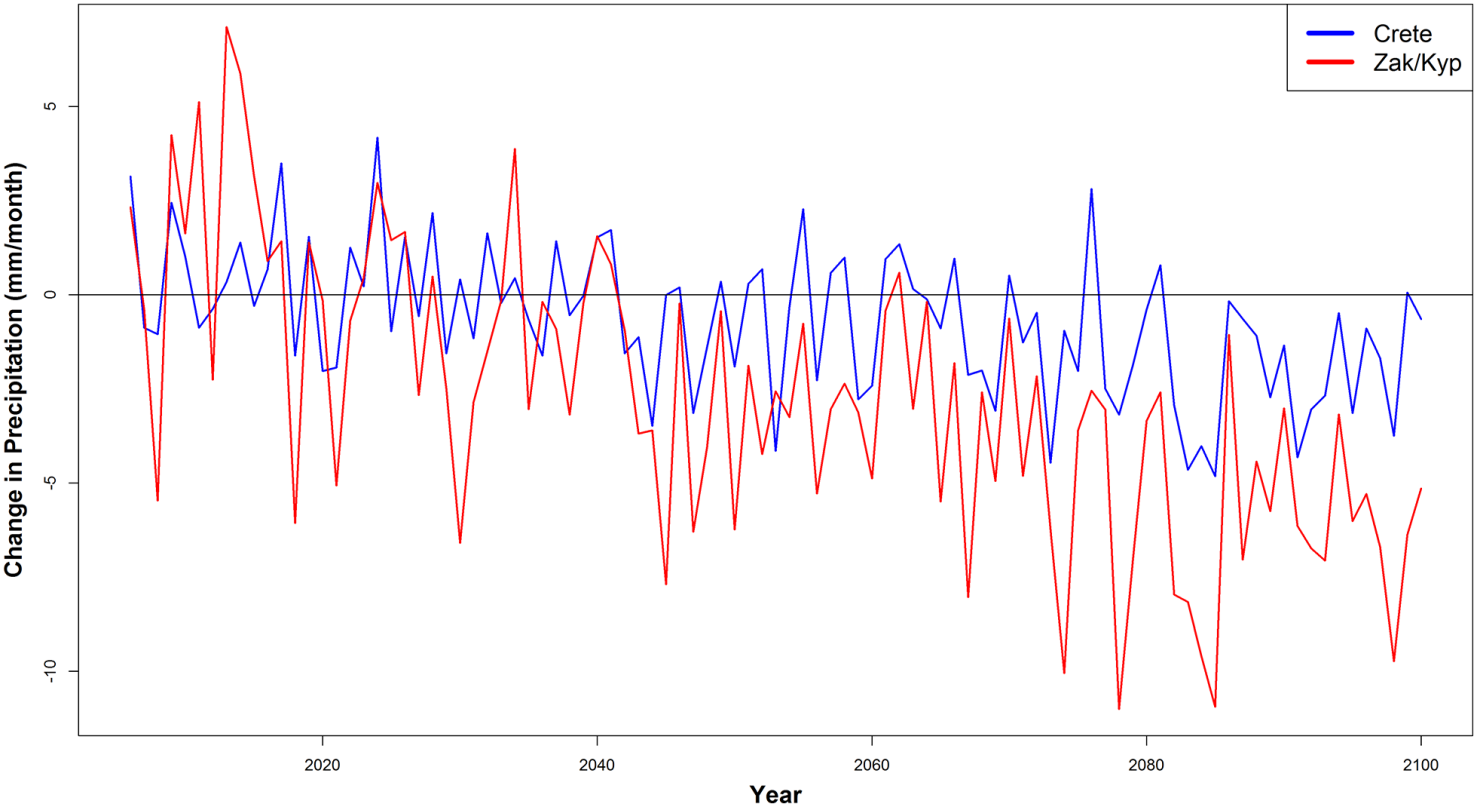
Means of the projected changes in precipitation rates from fourteen atmospheric models (RCP 8.5) during the mating season for Crete and Zakynthos/Kyparissia.

Such a shift, however, may not change nest success but could result in maintaining current levels. Climate model projections suggest that beach temperatures are expected to rise and precipitation is projected to decline (Fig 5), making future climate conditions in April and May similar to those during the current nesting season. As a result, even though loggerheads may shift nesting to earlier in the year, the climate conditions during nesting and hatching may remain the same. On the other hand, there is likely a seasonally constrained threshold, such that factors other than temperature may drive the timing of the nesting season (i.e. foraging area productivity, rainfall, number of nesting females per season) as has been demonstrated for other sea turtles [60].

We calculated, from the global climate models assessed by the IPCC, that SST at the breeding sites during April, May and June will become warmer by an average of 3.7°C. As a result, using a previously established relationship [27], the mean clutch sizes at these nesting beaches may be substantially reduced in the future. Recent results indicate the numbers are already declining (clutch size mean ± SD 2003–2009: Zakynthos: 106.7 ± 26.1 eggs) [6]. Based on models run by Mazaris et al. [27], the current SST at the breeding site of Zakynthos is just above the optimal condition to ensure a balance between smaller clutch sizes and a higher hatching success. A shift in phenology, as is expected for Zakynthos as temperatures increase, may help to offset the potential decline in clutch size, but changing conditions overall may have far-reaching effects on reproductive success.

Beach conditions, including sand temperature, precipitation, air temperature, bacterial load and proximity to vegetation also play key roles in the survival of hatchlings [16,20,24–26,61–63]. In a previous study, sand temperatures in Zakynthos and Kyparissia did not have a significant impact on hatching success [63], nor for loggerhead turtles nesting in Japan [64]. However, higher sand temperatures have been demonstrated to clearly impact hatching success of leatherback turtles (*Dermochelys coriacea*) [24,25]. Nests on Greek beaches had a particularly high hatching success (70–92%) compared to other loggerhead beaches throughout the world [6,33,63]. This may be due to the improved abiotic and biotic conditions of the beach environment, and reduced number of stochastic events, facilitating gas exchange in the nests [61,63]. Beach temperatures in Greece may also not yet have reached the thermal maximum for egg incubation (35°C; Ackerman [65]; 34°C; Moran et al. [66]), nor for hatchling emergence (32.4°C; Miller et al. [67]). On the beaches of Zakynthos and Kyparissia monitored respectively by Katselidis et al. [32] and Suss [63], nest temperature never exceeded the thermal maximum for incubation throughout the entire nesting and hatching seasons of 2008–2011 (Zakynthos) and 2010–2011 (Kyparissia). However, as global temperatures rise, this thermal maximum may be reached quite soon, as the highest nest temperature measured by Suss [63] was 33.8°C.

As sand temperatures warm and precipitation declines in the future, hatchling demographics could skew towards a more female bias. Such a skew, coupled with potential declines in hatching success and hatchling emergence rates, could play a strong role in reducing the overall nesting population for loggerheads in the future, as demonstrated in leatherbacks from the eastern Pacific [20]. In our study, within the sample period at Rethymno, sand temperatures at nest depth remained above the pivotal temperature for loggerheads in the region, beginning a month prior to the end of the nesting season. This temperature profile, that likely would lead to a strong female bias, corresponds with the existing trend for the Eastern Mediterranean of a strong skew towards a female sex ratio for loggerhead hatchlings within many of the monitored nesting beaches [32,68–75]. Currently, this trend does not yet seem to be manifesting as a strong female skew in the adult and later juvenile sex ratios [76,77], and long term historical models indicate the sex ratio in the past has most likely remained balanced [33]. However, a steady increase in sand temperatures could lead to stronger female biases in both primary and adult sex ratios in the future.

From our analyses, precipitation, historically, has not had a significant impact on nest success in Greece; however climate models project a substantial future reduction in annual precipitation at the nesting beaches. This may result in sand temperatures reaching the thermal maxima sooner, as well as sand moisture levels becoming too low for nesting [26,78]. Indeed, when taken all together, Saba et al. [20] projected climate change impacts on beach conditions would lead to a 7% decline per decade in leatherback turtles on a major nesting beach in Costa Rica, suggesting a possible trend for Mediterranean nesting beaches as well.

In previous studies [21,22], warmer SSTs at the foraging sites for loggerheads led to a reduction in nesting numbers on a two year lag. Similar trends were also found in green turtles (*Chelonia mydas*) in the South Pacific [40,79] and Eastern Pacific leatherbacks [19]. In the Mediterranean, Mazaris et al. [22] found that only a 1.5°C increase in SST at the foraging grounds corresponded to a decline of almost 500 nests per season in Zakynthos. We calculated a similar trend in Rethymno and found that the trend continued in Zakynthos when including more contemporary nesting data. Nesting declines may be due to the deterioration of the foraging grounds, but also may correspond to the strong anthropogenic impacts directly on these beaches, as this downward trend did not hold true for the nesting activity in Kyparissia Bay, a nesting beach with a much more limited human presence.

Nevertheless, as increases in SST limit available foraging resources, this may gradually reduce nesting output throughout the region. Greek loggerheads forage in regions typically characterized by shallow benthic environments with large areas of sea grass beds [10]. These sea grass meadows are essential for providing habitats for a very diverse set of species [80], including the benthic invertebrates preyed upon by loggerheads [43,44,81,82]. Undoubtedly, water temperature plays a crucial role in the survival of these sea grasses, specifically *Posidonia oceanica*, the most common sea grass in the Mediterranean [47,83,84]. Sea grass meadows already are steadily declining due to direct and indirect anthropogenic effects [46]. Furthermore, sea grasses are slow to recover from disturbances, and on the Spanish coast in the Western Mediterranean there is a negative net population growth rate of *Posidonia oceanica* [84]. A strong correlation also exists between the annual max SST and shoot mortality near the Balearic Islands, with an increase of 3°C causing shoot density to decline by approximately 13–40% along with an increase in mortality rate from 0.05 to 0.15 [47]. Over the last 30 years at loggerhead foraging sites, we found that SST during August, the warmest month of the year, increased by between 1.3–1.8°C and future projections of SST in August suggest a warming by another (mean ± SD) 4.4 ± 1.3°C by 2100. As a result, the sea grass meadows that loggerheads depend upon for much of their foraging may decline to 10% of their current density by as early as 2049 [48]. This potential reduction in food availability could have several impacts on loggerhead fitness, including reduced overall nesting output due to longer remigration intervals [39].

Climate plays a critical role in the survival of sea turtles both on the beach and at sea and additional data are required to improve our understanding of the connection between environmental features and sea turtle survival in this region. These include, more up to date data on regional nesting patterns and comprehensive assessments of the biotic and abiotic conditions of the critical nesting beaches. However, based on our analyses and those of related studies, as T_a_ and SST continue to rise and precipitation continues to decline, loggerhead turtles in the Mediterranean Sea may be able to temporarily compensate by nesting earlier in the season; however with an increasing skew in sex ratio, reduced soil moisture resulting in drier nest conditions, and the projected deterioration of the foraging grounds, minor phenological adjustments may not be enough to sustain the current populations. A combination of close examination of past and current data and our projections of future climate conditions suggests that Greek loggerhead populations will be threatened both at sea and at the nesting beaches if climate continues to warm in the Mediterranean region.

## Supporting Information

S1 Appendix Summary information and references for all of the climate change models used in this study. (PDF)

S2 Appendix Surface and nest depth sand temperatures during the 2012 nesting season for three beach sectors of Rethymno, Crete. (TIFF)

S3 Appendix Observed SST (1982–2012) during the mating season for Crete and Zakynthos/Kyparissia. (TIFF)

S4 Appendix Relationship between mating period SST and day of first female emergence at Zakynthos (p < 0.001, R2 = 0.830, y = -12.157(x) + 378.97). (TIFF)

S5 Appendix Observed and future projections for SST in August at the five high use regions. A) August SST at the 5 high use areas for loggerheads in the Mediterranean. Solid lines are the linear trend lines (Adriatic R^2^ = 0.046; Aegean R^2^ = 0.555; Crete R^2^ = 0.499; Gabès R^2^ = 0.190; Zak/Kyp R^2^ = 0.251). B) Means of the projected changes in August SST from 13 climate models (RCP 8.5) for the 5 high use sites.
(TIF)

S6 Appendix Observed T_a_ (1982–2012) during the mating season for Kyparissia, Rethymno and Zakynthos. (TIFF)

S7 Appendix Relationship between mating period T_a_ and day of first female emergence at Zakynthos (p < 0.01, df = 25, R2 = 0.705; y = -6.8327(x) + 286.35). (TIFF)
