# Supplementary material for: Climate Impacts on Sea Turtle Breeding Phenology in Greece and Associated Foraging Habitats in the Wider Mediterranean Region
Source: PLoS One. 2016 Jun 22;11(6):e0157170. doi: 10.1371/journal.pone.0157170 (PMC4917093; doi:10.1371/journal.pone.0157170)
Supplement: S1 Appendix — (PDF) [file pone.0157170.s001.pdf]

Model Name	Institution	Country	Atmosphere Model Resolution (lat x lon)	Ocean Model Resolution (lat x lon)	References
**ACCESS1.0**	Commonwealth Scientific and Industrial Research Organizaion and Bureau of Meteorology	Australia	1.25^o x 1.875^o	0.6^o x 1.0^o	[85]
**BCC-CSM1.1**	Beijing Climate Center, China Meteorological Administration	China	2.8^o x 2.8^o	0.8^o x 1.0^o	[86,87]
**CCSM4**	US National Center for Atmospheric Research	USA	1.0^o x 1.25^o	0.5^o x 1.125^o	[88,89]
**CMCC-CMS**	Centro Euro-Mediterraneo per I Cambiomenti Climatici	Italy	1.85^o x 1.875^o	1.2^o x 2.0^o	[90,91]
**CNRM-CM5**	Centre National de Recherches Meteorologiques and Centre Europeen de Recherche et Fromation Avancees en Calcul Scientifique	France	1.4^o x 1.4^o	0.6^o x 1.0^o	[92,93]
**CSIRO-Mk3.6.0**	Queensland Climate Change Centre of Excellence and Commonwealth Scientific and Industrial Research Organization	Australia	1.85^o x 1.875^o	1.0^o x 1.875^o	[94,95]
**FGOALS-g2**	LASG (Institute of Atmospheric Physics) - CESS (Tsinghua University)	China	4.7^o x 2.8^o	N/A	[96,97]

Model Name	Institution	Country	Atmosphere Model Resolution (lat x lon)	Ocean Model Resolution (lat x lon)	References
**GFDL-CM3**	NOAA Geophysical Fluid Dynamics Laboratory	USA	2.0^o x 2.5^o	0.9^o x 1.0^o	[98,99]
**GISS-E2-H**	NASA Goddard Institute for Space Studies	USA	2.0^o x 2.5^o	2.0^o x 2.5^o	[100]
**HadGEM2-AO**	National Institute of Meteorological Research/Korea Meteorological Administration	Korea	1.25^o x 1.875^o	1.0^o x 1.0^o	[101,102]
**INM-CM4**	Russian Institute for Numerical Mathematics	Russia	1.5^o x 2.0^o	0.5^o x 1.0^o	[103]
**IPSL-CM5A-MR**	Institut Pierre Simon Laplace	France	1.27^o x 2.5^o	N/A	[90,104]
**IPSL-CM5B-LR**	Institut Pierre Simon Laplace	France	N/A	1.2^o x 2.0^o	[90,105]
**MIROC5**	University of Tokyo, National Institute for Environmental Studies, and Japan Agency for Marine-Earth Science and Technology	Japan	1.4^o x 1.4^o	0.8^o x 1.4^o	[106]
**MRI-CGCM3**	Meteorological Research Insititute	Japan	1.121^o x 1.125^o	0.5^o x 1.0^o	[107]

85. Bi D, Dix M, Marsland SJ, O'Farrell S, Rashid H, Uotila P, et al. The ACCESS Coupled Model: Description, Control Climate and Evaluation. Aust. Meteorol. Ocean. 2013;63.1: 41 - 64.

86. Griffies SM, Gnanadesikan A, Dixon KW, Dunne JP, Gerdes R, Harrison A, et al. Formulation of an ocean model for global climate simulations. Ocean Sci. 2005;1: 45-79.

87. Xin X, Zhang L, Zhang J, Wu T, Fang Y. Climate change projections over East Asia with BCC_CSM1.1 climate model under RCP scenarios. J. Meteor. Soc. Japan, 2013;91: 413 – 429.

88. Danabasoglu G, Bates SC, Briegleb BP, Jayne SR, Jochum M, Large WG, et al. The CCSM4 Ocean Component. J. Clim. 2012;25: 1361-1389.

89. Neale RB, Richter J, Park S, Lauritzen PH, Vavrus SJ, Rasch PJ, Zhang M. The Mean Climate of the Community Atmosphere Model (CAM4) in Forced SST and Fully Coupled Experiments. J. Clim. 2013;26: 5150–5168.

90. Madec G, Delecluse P, Imbard M, Levy C. OPA 8.1 ocean general circulation model reference manual. IPSL Note du Pole de Modelisation, 1998. pp 91.

91. Manzini E, Cagnazzo C, Fogli PG, Bellucci A, Muller WA. Stratosphere-troposphere coupling at interdecadal time scales: Implications for the North Atlantic Ocean. Geophys. Res. Lett. 2012;39: L05801.

92. Madec G. NEMO ocean engine. Technical note. IPSL. Note du Pole de modélisation, Institut Pierre-Simon Laplace (IPSL), France, 2008. No 27 ISSN No 1288-1619.

93. Voldoire A, Sanchez-Gomez E, Salas y Melia D, Decharme B, Cassou C, Senesi S, et al. The CNRM-CM5.1 global climate model: description and basic evaluation. Clim. Dyn. 2013;40: 2091-2121.

94. Gordon H, O'Farrell S, Collier M, Dix M, Rotstayn L, Kowalczyk E, et al. The CSIRO Mk3.5 Climate Model. CAWCR Technical Report. 2010;21: 1-74.

95. Rotstayn LD, Jeffrey SJ, Collier MA, Dravitzki SM, Hirst AC, Syktus JI, Wong KK. Aerosoland greenhouse gas-induced changes in summer rainfall and circulation in the Australasian region: a study using single-forcing climate simulations. Atmos. Chem. Phys. 2012;12: 6377-6404.

96. Liu HL, Lin PF, Yu YQ, Zhang XH. The baseline evaluation of LASG/IAP Climate system Ocean Model (LICOM) version 2.0. Acta Meteorologica Sinica 2012;26: 318-329.

97. Li L, Lin P, Yu Y, Wang B, Zhou T, Liu L, et al. The flexible global ocean-atmosphere-land system model, Grid-point Version 3: FGOALS-g2. Adv. Atmos. Sci. 2013;30: 543 - 560.

98. Donner LJ, Wyman BL, Hemler RS, Horowitz LW, Ming Y, Zhao M, et al. The Dynamical Core, Physical Parameterizations, and Basic Simulation Characteristics of the Atmospheric Component AM3 of the GFDL Global Coupled Model CM3. J. Clim. 2011;24: 3484-3519.

99. Griffies SM, Greatbatch RJ (2012) Physical processes that impact the evolution of global mean sea level in ocean climate models. Ocean Model. 2012;51: 37-72.

100. Schmidt GA, Ruedy R, Hansen JE, Aleinov I, Bell N, Bauer M, et al. Present day atmospheric simulations using GISS ModelE: Comparison to in-situ, satellite and reanalysis data. J. Clim. 2006;19: 153-192.

101. Davies T, Cullen MJP, Malcolm AJ, Mawson MH, Staniforth A, White AA, Wood N. A new dynamical core for the Met Office's global and regional modelling of the atmosphere. Q. J. Roy. Meteor. Soc. 2005;131: 1759-1782.

102. Johns TC, Durman CF, Banks HT, Roberts MJ, McLaren AK, Ridley JK, et al. The new Hadley Centre Climate Model (HadGEM1): Evaluation of coupled simulations. J. Clim. 2006;19: 1327-1353.

103. Volodin EM, Dianskii NA, Gusev AV. Simulating present-day climate with the INMCM4.0 coupled model of the atmospheric and oceanic general circulations. Izv. Atmos. and Ocean. Phys. 2010;46: 414-431.

104. Hourdin F, Foujols MA, Codron F, Guemas V, Dufresne JL, Bony S, et al. Impact of the LMDZ atmospheric grid configuration on the climate and sensitivity of the IPSL-CM5A coupled model. Clim. Dyn. 2013a;40: 2167 – 2192.

105. Hourdin, F, Grandpeix JY, Rio C, Bony S, Jam A, Cheruy F, et al. LMDZ5B: the atmospheric component of the IPSL climate model with revisited parameterizations for clouds and convection. Clim. Dyn. 2013b;40: 2193-2222.

106. Watanabe M, Suzuki T, O'ishi R, Komuro Y, Watanabe S, Emori S, et al. Improved Climate Simulation by MIROC5: Mean States, Variability, and Climate Sensitivity. J. Clim. 2010;23: 6312-6335.

107. Yukimoto S, Adachi Y, Hosaka M, Sakami T, Yoshimura H, Hirabara M, et al. A New Global Climate Model of the Meteorological Research Institute: MRI-CGCM3-Model Description and Basic Performance. J. Meteor. Soc. Japan. 2012;90A: 23-64.
