# Supplementary figures and images for: Climate Impacts on Sea Turtle Breeding Phenology in Greece and Associated Foraging Habitats in the Wider Mediterranean Region
Source: PLoS One. 2016 Jun 22;11(6):e0157170. doi: 10.1371/journal.pone.0157170 (PMC4917093; doi:10.1371/journal.pone.0157170)

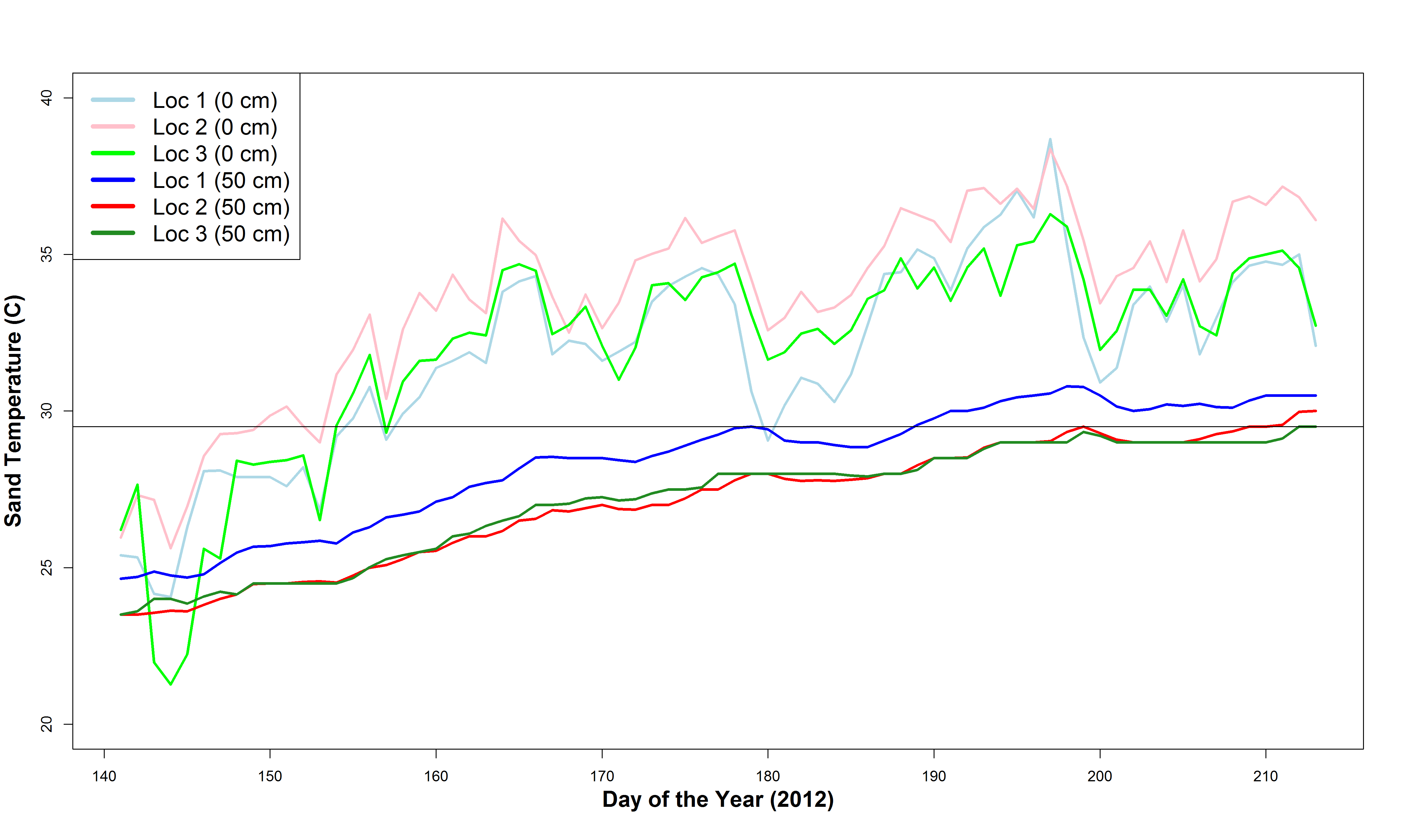

Supplement: S2 Appendix — (TIFF) [file pone.0157170.s002.tiff]

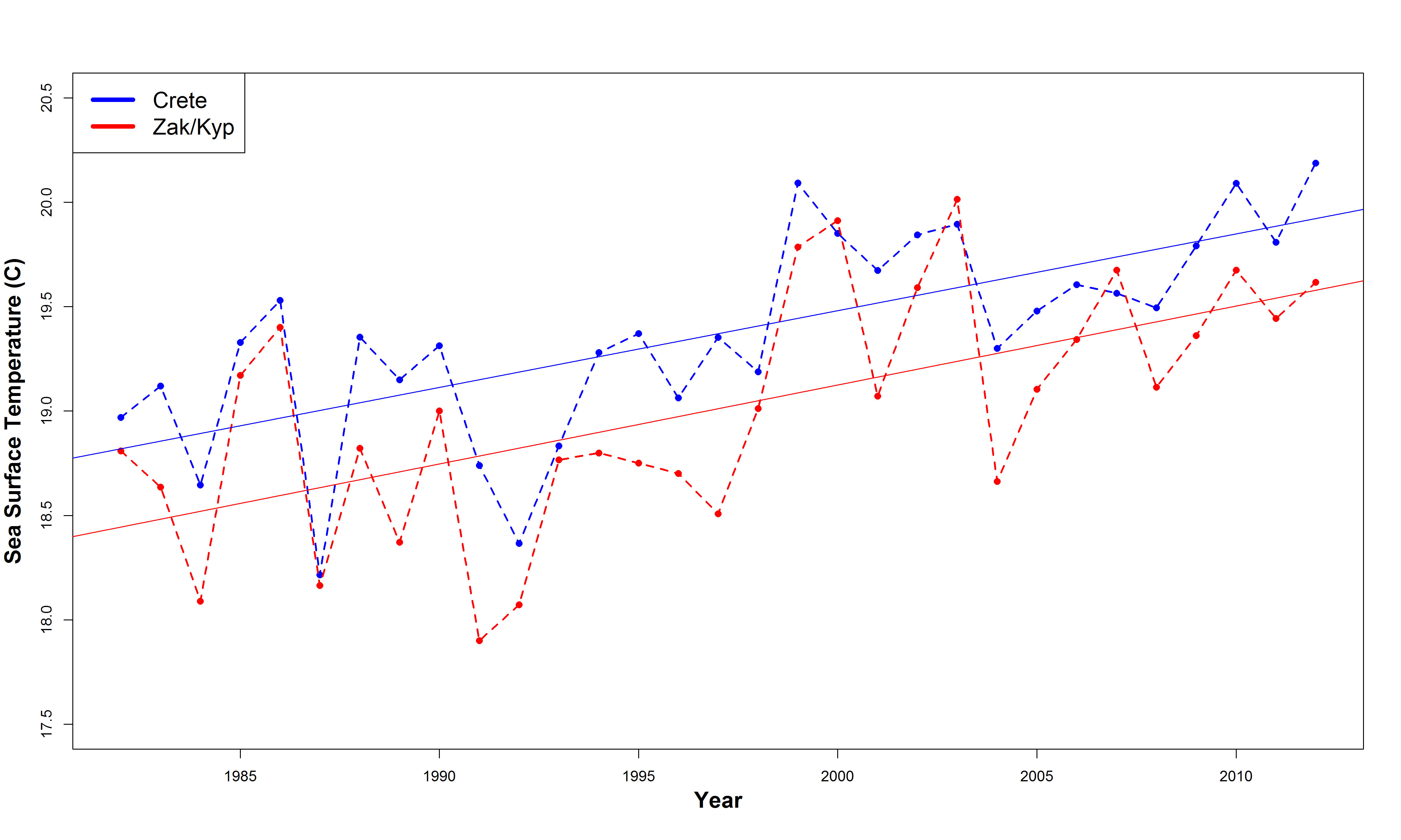

Supplement: S3 Appendix — (TIFF) [file pone.0157170.s003.tiff]

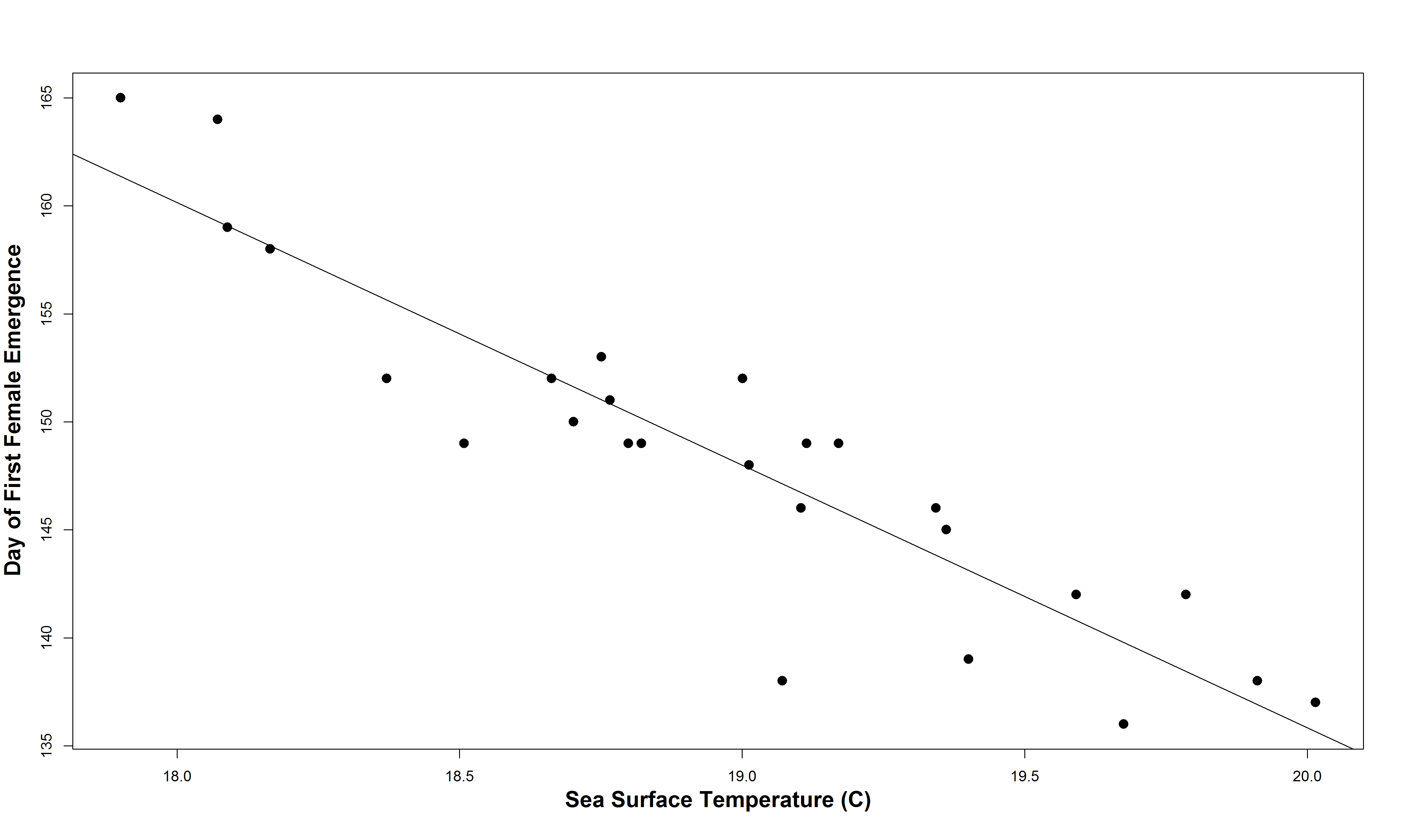

Supplement: S4 Appendix — (TIFF) [file pone.0157170.s004.tiff]

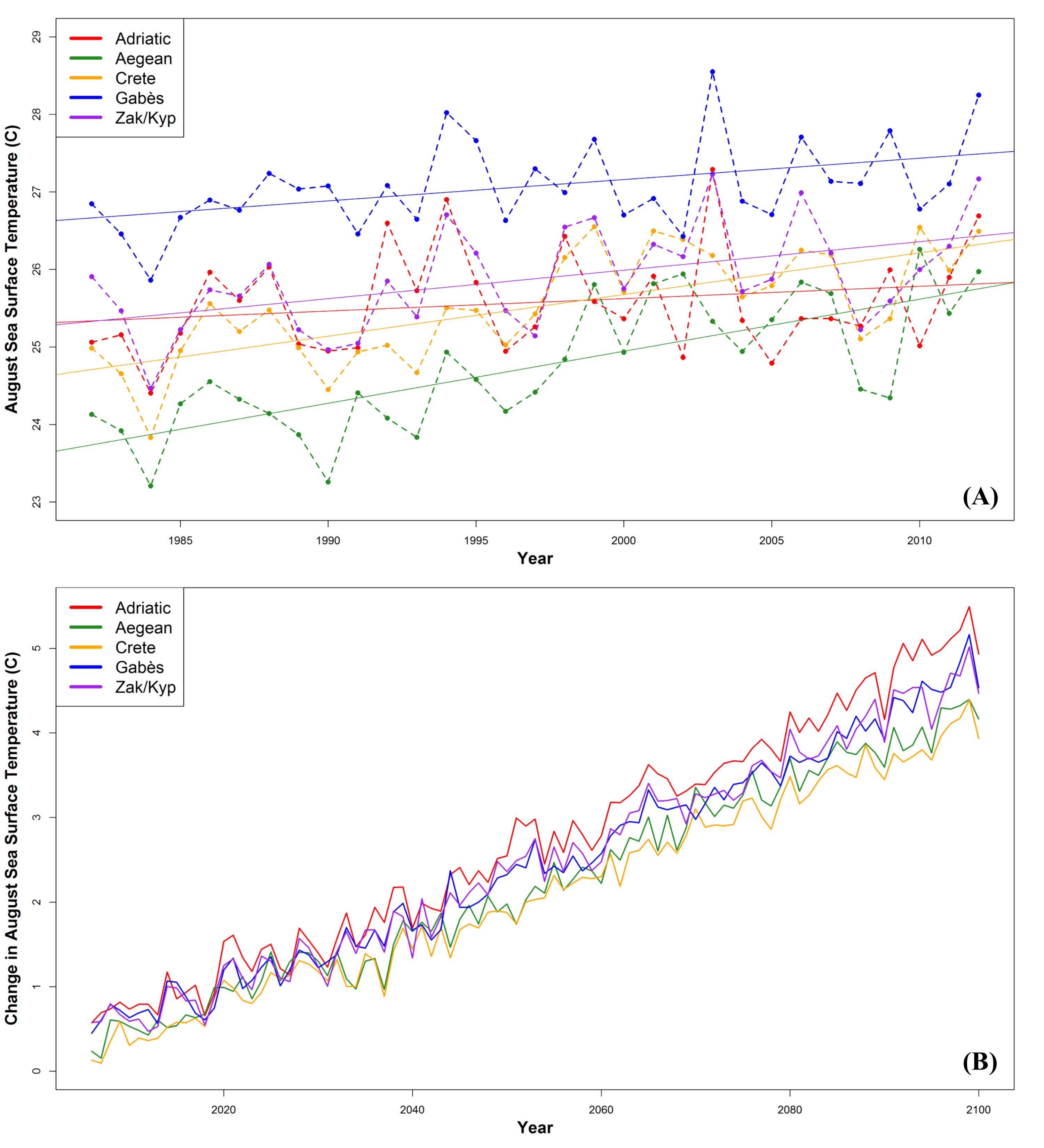

Supplement: S5 Appendix — A) August SST at the 5 high use areas for loggerheads in the Mediterranean. Solid lines are the linear trend lines (Adriatic R2 = 0.046; Aegean R2 = 0.555; Crete R2 = 0.499; Gabès R2 = 0.190; Zak/Kyp R2 = 0.251). B) Means of the projected changes in August SST from 13 climate models (RCP 8.5) for the 5 high use sites. (TIF) [file pone.0157170.s005.tif]

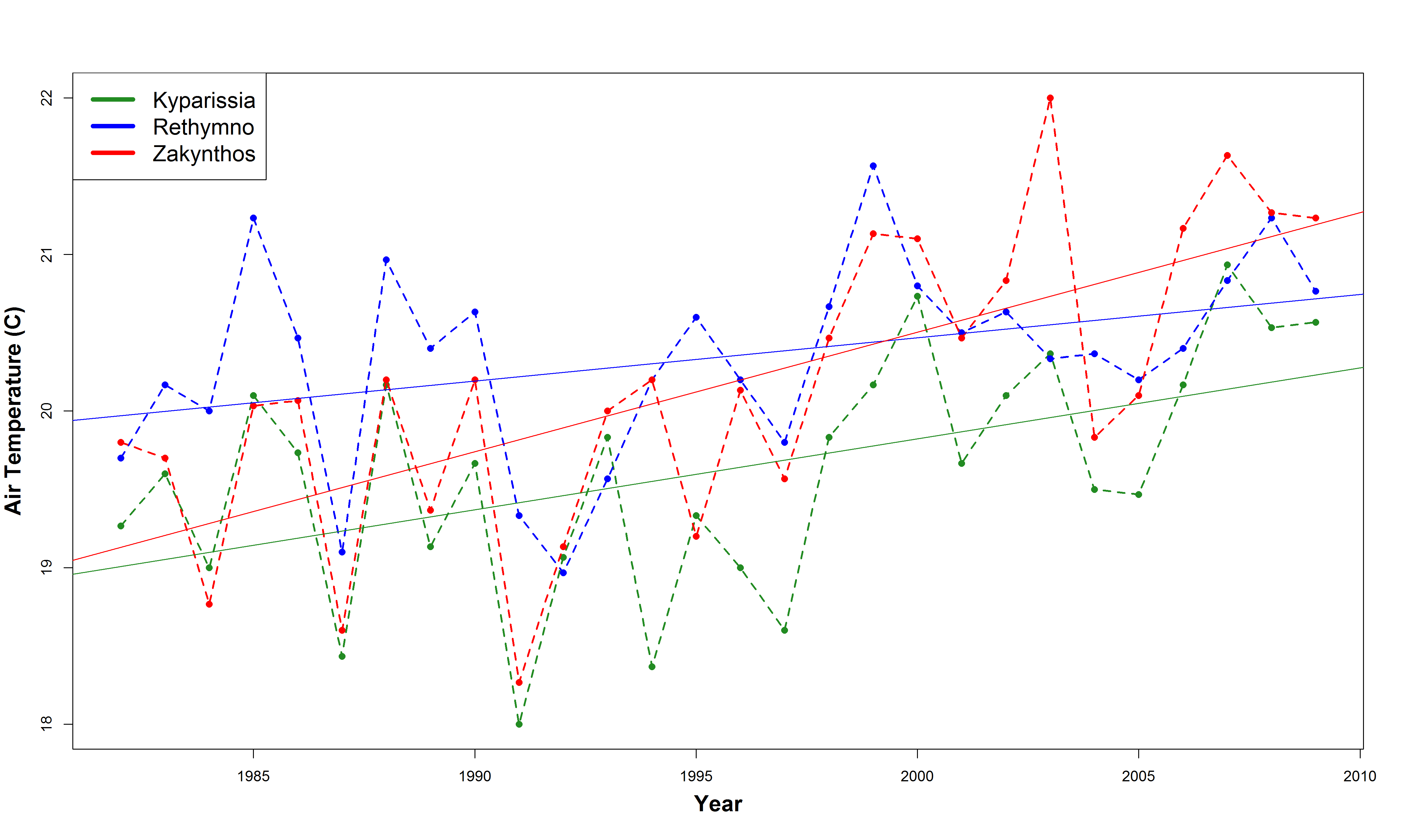

Supplement: S6 Appendix — (TIFF) [file pone.0157170.s006.tiff]

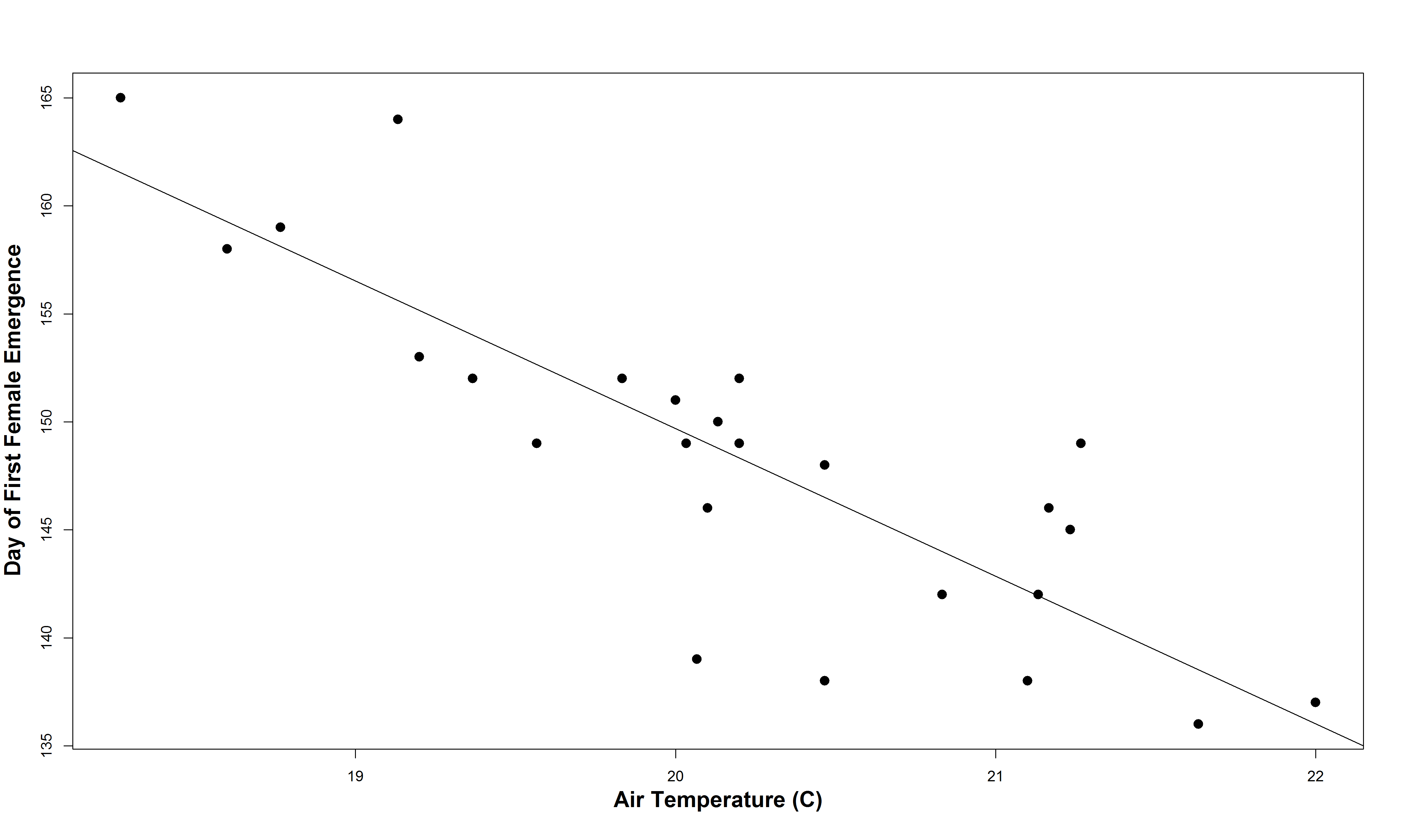

Supplement: S7 Appendix — (TIFF) [file pone.0157170.s007.tiff]
